# ﻿*Viola yuelingensis* (Violaceae), a new species from Guangxi, China

**DOI:** 10.3897/phytokeys.267.172198

**Published:** 2025-12-08

**Authors:** Ju-Hua Huang, Jian-Hua Zhang, You-Sheng Chen

**Affiliations:** 1 College of Tourism and Landscape Architecture, Guilin University of Technology, Guilin 541006, Guangxi, China; 2 Guangxi Key Laboratory of Plant Conservation and Restoration Ecology in Karst Terrain, Guangxi Institute of Botany, Guangxi Zhuang Autonomous Region and Chinese Academy of Sciences, Guilin 541006, Guangxi, China; 3 Guangxi Guilin Mao'er Mountain National Nature Reserve Administration, Guilin 541000, Guangxi, China; 4 South China Botanical Garden, Chinese Academy of Sciences, Guangzhou 510650, China; 5 Center of Conservation Biology, Core Botanical Gardens, Chinese Academy of Sciences, Guangzhou 510650, China

**Keywords:** Morphology, new species, taxonomy, *
Viola
*

## Abstract

This study describes and illustrates *Viola yuelingensis* Y.S.Chen & Ju H.Huang, a new species distributed in Guangxi, China. The species is morphologically most similar to *Viola yunnanensis*, but can be distinguished by the following characteristics: prominently raised reddish-brown midveins on both leaf surfaces with inconspicuous lateral veins on the adaxial side (vs. wrinkled adaxial surface with depressed and often green midvein), and lower petal white or pale purple toward the base, pale purple toward the apex with purple stripes (vs. lower petal white with purple stripes). The species is distinct from *V. changii* by its leaf blades with serrate margins (vs. obtusely serrate to subentire), and an abaxial surface that is grayish-green (vs. deep purple). Color photographs, a distribution map, and a comparison with the most similar species are provided.

## ﻿Introduction

The Violaceae Batsch is a family of core eudicot plants comprising approximately 22 genera and more than 900 species, with a widespread global distribution. The Violaceae is represented by four genera and an estimated 120 species in China according to Flora of China (Wu et al. 1994). These taxa are widely distributed across the country, encompassing temperate northern zones, subtropical and tropical southern regions (Li et al. 2022). The majority of species are distributed in southwestern China, followed by significant diversity in the northeastern and northern parts of the country.

According to current understanding, taxonomic gaps remain in the classification of Violaceae species distributed across southern and southwestern China, indicating that new species await discovery (Ning et al. 2012). Over the past five years, several new Violaceae species from China have been described, including *Viola austroyunnanensis* Y.S.Chen (Liao et al. 2023), *Viola xinchengensis* You Nong & G.Y.Wei (Wei et al. 2025), *Viola qingruii* Yan S.Huang & Q.Fan, *Viola heyuanensis* Yan S.Huang, Q.L.Ye & Q.Fan, *Viola chaozhouensis* Yan S.Huang, J.H.Ding & Q.Fan, *Viola longissima* Yan S.Huang & Q.Fan (Huang et al. 2023b), *Viola shiweii* Xiao C.Li & Zheng W.Wang (Li et al. 2022), *Viola pendulipes* Yan S.Huang & Q.Fan (Huang et al. 2023a), and *Viola huizhouensis* Y.S.Huang & Q.Fan (Huang et al. 2021).

Guangxi, recognized as a biodiversity hotspot, has continually revealed new species or new species records during annual field investigations (Feng et al. 2021). Since 2024, we have conducted consecutive field surveys in Guilin City, Guangxi, collecting numerous specimens of Violaceae. Each specimen was carefully examined and identified, with consultations of experts in relevant fields for challenging taxa. In April 2025, during field surveys in Xing’an County and Lingchuan County of Guilin City, we discovered a distinct *Viola* population. While its habit, floral and fruit morphology resemble that of *Viola yunnanensis*, this taxon exhibits several differentiating characteristics: sparsely pubescent throughout, stipules mostly free with fimbriate-dentate margins, serrate leaf margins, prominently raised and reddish-brown midveins on both adaxial and abaxial leaf surfaces, pale purple flowers, and a very short spur.

After nearly a year of field tracking, supplemented by detailed morphological dissection, comparison, and validation, we confirmed that this morphologically distinct *Viola* population represents a new species.

## ﻿Material and methods

Field surveys and observations were conducted on the flowering and fruiting periods of the presumed new species. Additionally, specialists in Violaceae taxonomy were consulted to assist with specimen identification. Fresh and dried specimens were examined under microscopes and stereo microscopes to document morphological characteristics. The specimens have been deposited at the Herbarium (**IBK**), Guangxi Institute of Botany, Guangxi Zhuang Autonomous Region and Chinese Academy of Sciences.

During the flowering and fruiting period (March to September), we conducted field observations of living specimens of this species. After collecting floral and fruit samples, we transported some of the plants back to Guilin Botanical Garden in Guangxi for cultivation. Upon completion of the dissection, comprehensive morphological documentation was conducted, encompassing stem architecture, foliar features, pedicel structure, floral morphology, receptacle form, petal details, stamen arrangement, pistil anatomy, and capsule fruit characteristics. We conducted detailed observations and measurements of plant structures including style, ovary, and stipules using a tape measure, caliper, and a microscope with magnification over 10×.

The description of this new species is based on field observations and herbarium specimen studies from 2025. The identification of other related species in the genus was referenced to online images from the Flora of China catalog (www.iplant.cn/frps) and JSTOR Global Plant Database (http://plants.jstor.org/), combined with specimens from the Digital Specimen Museum of China (https://www.cvh.ac.cn/index.php). Supplementary information regarding collection sites, habitats, plant morphology, and fruit details was compiled through field surveys and specimen label references. In accordance with the International Union for Conservation of Nature (IUCN) 2022 classification standards, we assessed the provisional conservation status of newly discovered species.

## ﻿Results and discussion

*Viola yuelingensis* and *V. changii* are allopatric and morphologically distinct. *V. changii* is currently only known from Guangdong, Fujian, Hunan and Jiangxi province in China, where it inhabits rocky crevices at forest margins and moist sites within evergreen broad-leaved forests (according to the image data from the Plant Photo Bank of China). In contrast, *V. yuelingensis* is currently known only from Xing’an and Lingchuan Counties in Guangxi. *Viola* species are commonly found in soils or rock crevices within open areas along valley edges whereas *V. yuelingensis* predominantly inhabits slightly acidic soils under montane forests, karst limestone formations, and rocky cliffs along gullies in Northern Guangxi, China. On this mountain, researchers did not observe any intermediate forms of this species. Its significant morphological distinctions from the primary comparative species, *V. yunnanensis* and *V. changii*, further support its status as a well-differentiated species.

The phylogeny reconstructed from complete ITS sequences is insufficient to clarify the relationships among its subgenera or taxa, However, it offers valuable insights for the systematics of lower-level *Viola* taxa, as the composition and relationships of most sections are well corroborated by morphological evidence (Gong et al. 2010). Applying these molecular techniques to clarify the phylogenetic placement and infrageneric relationships of *V. yuelingensis* represents a logical next step.

### ﻿Taxonomic treatment

#### ﻿ Viola yuelingensis

Taxon classificationPlantaeMalpighialesViolaceae

Y.S.Chen & Ju H.Huang sp. nov.

596BF0CF-B2D5-56E7-9051-7C09DE27DA7C

urn:lsid:ipni.org:names:77372983-1

Figs 1, 2

##### Diagnosis.

This species’ creeping stems typically exhibit a dark red coloration with white pubescence. The leaves are ovate or ovate, featuring a narrow, shallowly cordate base, a pointed or gradually tapering apex, and serrated margins covered with white hairs. The reddish-brown midvein protrudes prominently on both leaf surfaces. Petals range from pale purple to white, with lower petals bearing rod-like appendages at their bases and narrow, flattened margins along the style’s upper end. Mature seeds display a distinctive yellowish-brown hue, making this species easily distinguishable from other members of the genus. It differs from *Viola yunnanensis* in several key features: reduced overall hairiness, serrated leaf margins, reddish-brown midveins with raised leaf surfaces, indistinct lateral veins on upper leaves, lanceolate or cordate stipules (0.5–2 cm long, reddish-brown or greenish) with elongated fringed teeth at tips, calyx segments lacking appendages at base ends, and lower petals showing white or pale purple near the base, with purple stripes near the apex (shown in Fig. 3). *Viola yuelingensis* is similar to *V. changii*, but it can be easily distinguished by its leaves which are 3–5 cm long and 2–4 cm wide, with serrate margins (vs. leaves are 1.5 cm long and 1.2 cm wide, with obtusely serrate to subentire margins) and the leaf blade is green adaxially and grayish-green or pale green abaxially, with a reddish-brown midvein (vs. the adaxial surface is dark green and densely villous, while the abaxial surface is deep purple). More detailed morphological differences amongst the three similar species are shown in Table 1.

**Table 1.** Main morphological differences amongst *Viola yuelingensis*, *V. yunnanensis* and *V. changii*.

Characters	*Viola yuelingensis* sp. nov.	*Viola yunnanensis*	*Viola changii*
Stipules	lanceolate or rhombic, 0.5–2 cm long, reddish brown or greenish, with a gradually pointed tip, membranous, and with long fringe teeth on the edges	lanceolate, 1–1.3 cm long, pale white, with a gradually pointed tip, membranous, and with long fringe teeth on the edges	lanceolate, light green, tapering at the apex, with long serrations along the margins, covered with white soft hairs
Leaf blade	ovate to broad-ovate, 3–5 cm long and 2–4 cm wide; base narrowly cordate; margins serrate; midvein reddish-brown, prominent on both surfaces; sparsely hairy on both side	oblong to oblong-ovate, 3–8 cm long, 2–4 cm wide, broadest near middle; apex acute or acuminate; base shallowly and narrowly cordate; margin coarsely crenate; both surfaces densely grayish-white pubescent	ovate or ovate orbicular, 1.5 × 1.2 cm, margin obtusely crenate or subentire, abaxially dark purple and puberulous only along veins but adaxially dark green and densely puberulous
Sepals	narrow-lanceolate, reddish or greenish, base elevated or sessile, sparsely pubescent	green, linear-lanceolate or lanceolate, with short basal appendages and 3 veins, with sparse white hairs along the veins and dense marginal hairs on the margins	broad lanceolate, brown or green, base elevated, velvety hairs on the surface
Flowers	corolla pale purple, pale blue, to white; petals obovate to oblong	flowers white to pinkish; petals oblong	flowers white to light purple, base green; petals oblong
Side petals	sparsely bearded	not bearded at the base inside	sparsely bearded
Lower petals	proximal base is white or pale purple, and the proximal tip is pale purple with purple stripes	white with purple stripes	pale purple with purple stripes, the lower petal is obtuse
Ovary	cylindrical, style rod-shaped, slightly curved at the base	oval, style rod-shaped, nearly erect at the base	cylindrical, style rod-shaped, curved at the base

**Figure 1.**
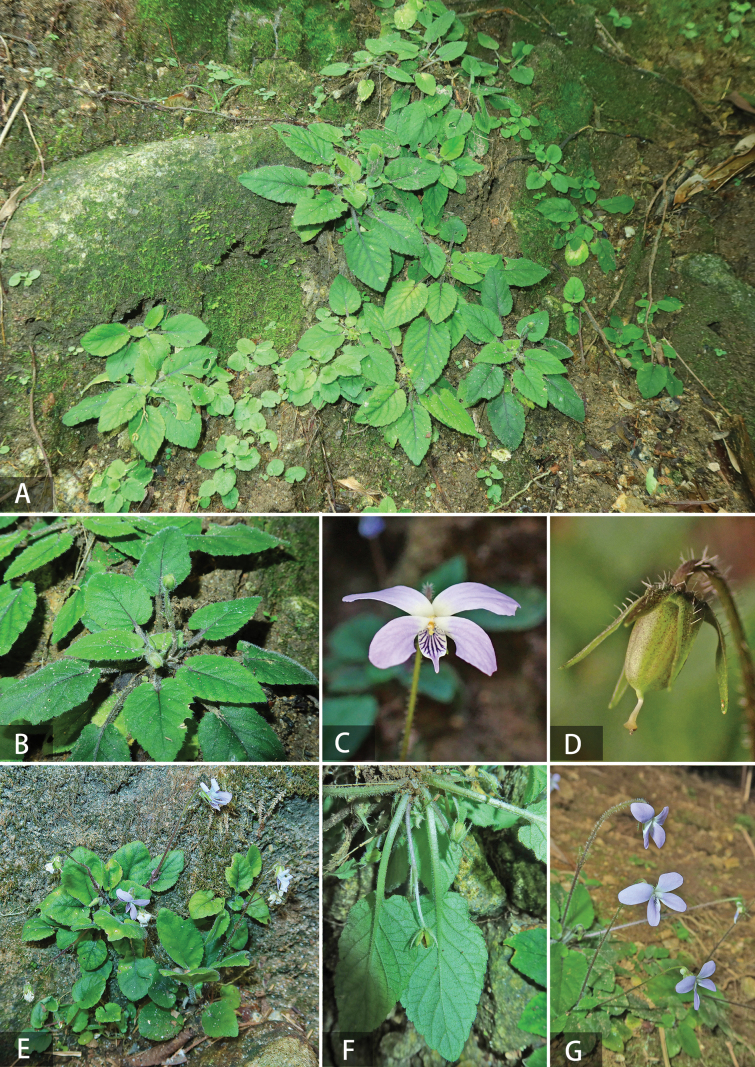
*Viola yuelingensis* Y.S.Chen & Ju H.Huang. A. Understory habitats; B. Individual plant; C. Flowers in front view; D. Fruit side; E. The habitat on the stone wall; F. Back of leaf; G. Inflorescence. Images quoted from type specimens MB3651 & MA3117 (IBK).

**Figure 2.**
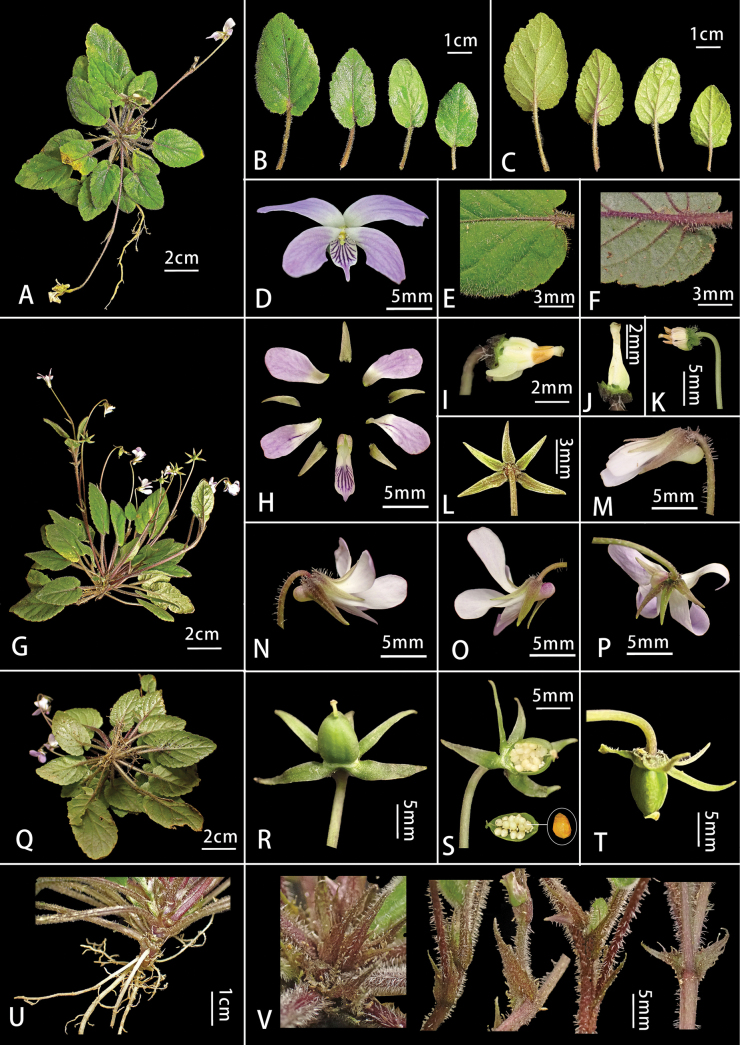
*Viola yuelingensis* Y.S.Chen & Ju H.Huang. A. Top view of plant; B. Leaf in front view; C. Back of leaf; D. Flowers in front view; E, F. Leaf details; G. Vertical plan of the plant; H. Floral anatomy; I. Stamen and pistil; J. Ovary; K. Stamen and pistil; L. Sepals; M. Side of bud; N, O. Floral side; P. Back of the flower; Q. Back view of plant; R. Fruit in front view; S. Fruit longitudinal section; T. Fruit side; U. Root; V. Stipules. Images quoted from type specimens MB3651 (IBK).

**Figure 3.**
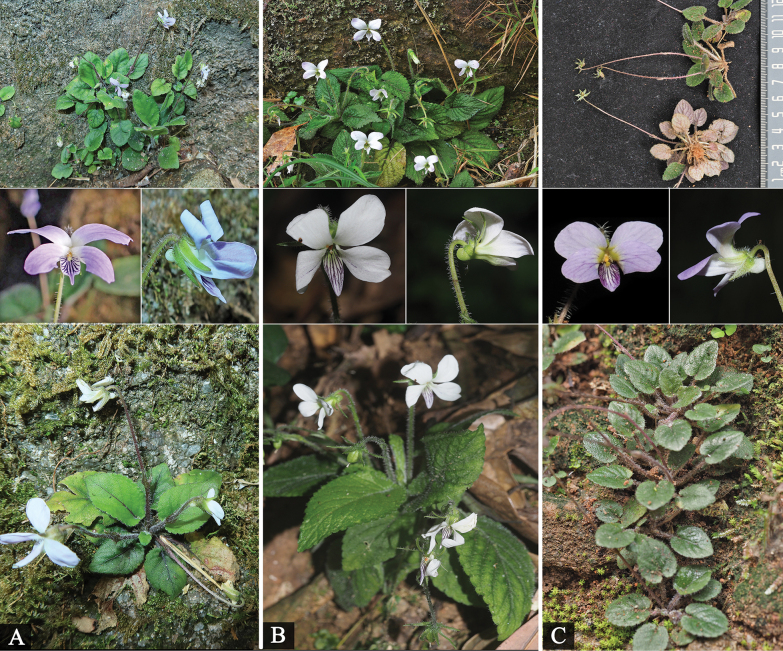
*Viola yuelingensis* Y.S.Chen & Ju H.Huang, *V. yunnanensis* and *V. changii*. A. *V. yuelingensis*; B. *V. yunnanensis*; C. *V. changii* (*V. yunnanensis* and *V. changii* photographed by You-sheng Chen, voucher specimen information: *V. yunnanensis*, China, Yunnan Province, Yuanyang County, Daping Township, Dayutang Village, 1441 m, 1 April 2020, *You-sheng Chen et al. YY1167* (IBSC); *V. changii*, China, Guangdong Province, Renhua County, Danxia Mountain, Yuxiu Pavilion-Jiulong Pavilion, 25°02'N, 113°73'E, alt. 121 m, 3 April 2021, *You-sheng Chen & Zi-Chao Jin*, *SG01* (IBSC); *V. yuelingensis*, China, Guangxi, Guilin City, Xing’an County, Huajiang Township, Dazhushan, Yangque Ridge Village, 25°84'N, 110°44'E, alt. 966 m, in the cool and humid undergrowth of the hillside forest, 19 April 2025, *Ju-Hua Huang et al. MA3117* (IBK).

##### Type.

China • Guangxi, Guilin City, Xing’an County, Huajiang Township, Jiejiaowan Village to Yapo Mountain, coordinates: 25.78, 110.41, elevation 751.73 meters, understory of hillsides and along riverbanks in crevices, 27 July 2025, *Ju-Hua Huang et al. MB3651* (holotype IBK00472880; isotypes IBK00472881, IBK00472882, IBK00472883).

##### Description.

Perennial herb, acaulescent, with an elongated rhizome bearing nodes. Rhizome grows obliquely or horizontally along the ground forming stolons (sometime absent) with adventitious roots at its nodes. Stolons slender, often reddish-brown, measure 1–40 cm in length densely covered with white pubescence, producing new shoots from alternate or clustered leaflets at the tips. Leaves grow near the base or alternate along the stolons, presenting ovate, broadly ovate, or ovate shapes (3–5 × 2–4 cm), broadest at or below the middle, acute or gradually tapering tips, shallow cordate bases, margin serrate, grayish-white pubescent on both surfaces. Surface of leaves dark green to dark green, while the lower surface shows grayish-green or pale green coloration. Midvein reddish-brown, prominent abaxially and adaxially; lateral veins faint. Petioles 3–5 cm, often reddish-brown, densely pubescent. Leaves mostly freelance, lanceolate or cordate stipules (0.5–2 cm long), attenuate at apex. Stipules feature elongated fringelike teeth along margins, appearing reddish-brown or greenish, membranous texture. Flowers 1.5 cm in diameter, corolla pale purple, light blue, or white colors, petals 5, differentiated into three types: upper petal (2), lateral petals (2), and lower petal (1). Upper petal obovate or oblong, nearly symmetrical, 0.5–1.5 × 0.5–1 cm. Lateral petals similarly obovate or oblong, 0.5–1.2 × 0.3–0.5 cm, white bases and sparsely bearded with curved inward club-like appendages. Lower petal oblong or ovate, 0.5–1.5 × 0.5–1 cm, apex gradually acuminate, base tapers to white or pale purple, distal portion pale purple with distinct purple striations. Spur short, saccate, 2–2.5 × ca. 2 mm, arises from the lower petal base; spur of 2 anterior stamens bear angular, acutely tipped spurs. Ovary cone-shaped, glabrous, featuring a club-shaped style, curves upward at base. Styles narrow, flat margin and a short beak terminating in an upward-opening pore, with a stigma hole open upward at tip of beak. Pedicel 4–12 cm long, often exserted beyond foliage, rufous with dense white pubescence; bearing two linear bracts inserted oppositely at mid-length. Sepals narrowly ovate-lanceolate or lanceolate, 3–7 mm long, gradually tapering to points, convex or sessile, with soft hairs along margins and veins. Capsule oblong or trigonous, 5–7 mm long, green, splitting into three segments when mature. Seeds spherical or oval, turning yellowish-brown upon maturity.

##### Phenology.

Flowering occurs from March to July, with fruiting lasting from April to September.

##### Etymology.

The specific term ‘*yueling*’ specifically refers to its model origin, China Guangxi, yuechengling.

##### Distribution and habitat.

The new species is known only from Guangxi Xing’an County and Lingchuan County of Guilin City. It grows in moist areas such as mountainous woodlands, forest-edge grasslands, valleys, or rocky cliffs along roadsides at altitudes ranging from 200 to1000 meters.

##### IUCN Red List Category.

Data available for the new species are still insufficient to assess its conservation status. According to the IUCN Criteria (IUCN 2022), it is considered Data Deficient (DD) until more information becomes available. Although *Viola yuelingensis* currently has relatively good growth, further collection and monitoring are necessary to allow more conclusive estimations about the rarity and vulnerability of the species.

##### Additional specimens examined (paratypes).

China • Guangxi, Guilin City, Xing’an County, Huajiang Township, Longtanjiang Scenic Area, Sandian River, 25°81'N, 110°42'E, alt. 643 m, on the stone wall beside the hillside ravine and under the forest, 21 April 2025, *Ju-Hua Huang et al. MA3166* (IBK!); • Guangxi, Guilin City, Xing’an County, Huajiang Township, Dazhushan, Yangque Ridge Village, 25°84'N, 110°44'E, alt. 966 m, in the cool and humid undergrowth of the hillside forest, 19 April 2025, *Ju-Hua Huang et al. MA3117* (IBK!); • Guangxi, Guilin City, Lingchuan County, Lantian Township, beside Township Road 175 near Tanxia Town, 25°54'N, 110°16'E; alt. 295 m, beside the hillside road, 21 March 2025, *Ju-Hua Huang et al. MA3002* (IBK!); • Guangxi, Guilin City, Lingchuan County, Jiuwu, Niulujie, 300 m, 1 April 2025, *Yun-Biao Liao & Ju-Hua Huang CYS25014* (IBSC).

##### Taxonomic notes.

*Viola yuelingensis* produces creeping stolons and rhizomes. The ovary is conical and glabrous, while the column is clavate with a slightly curved, gradually thickening base. The column apex is smooth and shallowly emarginate. Lateral petals are bearded. Peduncles are covered with patent hairs (rarely glabrous). The rhizome is short and densely noded. Stolons bear 1–2 reduced leaves and terminate in a leaf rosette. The corolla is typically pale pink to pale violet with a greenish throat. According to the taxonomic revision of *Viola* by Marcussen et al. (2022), morphological analysis confirmed that the new species belongs to Viola sect. Plagiostigma subsect. Diffusae W. Becker.

## Supplementary Material

XML Treatment for Viola yuelingensis

## ﻿Additional information

### Use of AI

No use of AI was reported.

### Author ORCIDs

Ju-Hua Huang https://orcid.org/0009-0003-4002-2554

Jian-Hua Zhang https://orcid.org/0009-0007-9717-4996

You-Sheng Chen https://orcid.org/0000-0002-7729-1075

### Data availability

All of the data that support the findings of this study are available in the main text.
